# A Single Bout of High-Intensity Cardiovascular Exercise Does Not Enhance Motor Performance and Learning of a Visuomotor Force Modulation Task, but Triggers Ipsilateral Task-Related EEG Activity

**DOI:** 10.3390/ijerph182312512

**Published:** 2021-11-27

**Authors:** Nils Henrik Pixa, Lena Hübner, Dieter F. Kutz, Claudia Voelcker-Rehage

**Affiliations:** 1 Department of Neuromotor Behavior and Exercise, Institute of Sport and Exercise Sciences, University of Münster, 48149 Münster, Germany; dieter.kutz@uni-muenster.de (D.F.K.); claudia.voelcker-rehage@uni-muenster.de (C.V.-R.); 2 Institute of Human Movement Science and Health, Faculty of Behavioral and Social Sciences, Chemnitz University of Technology, 09107 Chemnitz, Germany; huebner.lena@web.de

**Keywords:** physical exercise, fine motor control, motor learning, alpha oscillations, beta oscillations, EEG power, Bayesian ANOVA

## Abstract

Acute cardiovascular exercise (aCE) seems to be a promising strategy to improve motor performance and learning. However, results are heterogeneous, and the related neurophysiological mechanisms are not well understood. Oscillatory brain activitiy, such as task-related power (TRPow) in the alpha and beta frequencies, are known neural signatures of motor activity. Here, we tested the effects of aCE on motor performance and learning, along with corresponding modulations in EEG TRPow over the sensorimotor cortex. Forty-five right-handed participants (aged 18–34 years) practiced a visuomotor force-matching (FM) task after either high-intensity (HEG), low-intensity (LEG), or no exercise (control group, CG). Motor performance was assessed immediately, 15 min, 30 min, and 24 h after aCE/control. EEG was measured during the FM task. Results of frequentist and Bayesian statistics revealed that high- and low-intensity aCE had no effect at the behavioral level, adding to the previous mixed results. Interestingly, EEG analyses showed an effect of aCE on the ipsilateral sensorimotor cortex, with a stronger decrease in β-TRPow 15 min after exercise in both groups compared to the CG. Overall, aCE applied before motor practice increased ipsilateral sensorimotor activity, while motor learning was not affected; it remains to be seen whether aCE might affect motor learning in the long run.

## 1. Introduction

Acquiring, maintaining, and restoring motor skills is essential to accomplishing daily activities at home, at work, and during recreation, and allowing people to lead independent lifestyles. Therefore, it is of utmost interest to investigate strategies that promote processes of motor skill learning and support motor skill maintenance in several settings, such as in neurorehabilitation and healthy aging, in work-related fields such as neuroergonomics, and in other areas, such as elite sports and professional musicians.

In recent years, a growing body of research has investigated whether motor performance and learning can be enhanced by even a single bout of acute cardiovascular exercise (aCE; for reviews, see [1,2,3,4]). In particular, high-intensity aCE (>60% VO_2_-max, or >70% of the HR-max) has previously been shown to improve motor skill learning [5,6,7,8,9,10], and a recent meta-analysis indicated that it is more effective in comparison to lower intensity exercise [4].

The positive effects of aCE on neuroplasticity are thought to be related to several neurophysiological mechanisms. In this way, it is thought that exercise might act as a kind of primer to create a favorable environment for neuroplastic processes of motor learning [8,11,12] (see [13,14] for reviews); however, such exercise-induced neuromodulations on several neurophysiological system levels are yet not fully understood. Further insights into the neuroplastic mechanisms associated with the effects of aCE might be gained by investigating electrocortical signatures of motor performance and learning, as assessed via electroencephalography (EEG), which can be performed concurrently with the practiced motor task.

In general, motor performance relies on the activity of widespread cortical and subcortical networks, and motor skill learning is accompanied by neuroplasticity within these motor networks. These networks are made up of the primary motor (M1) and sensory cortex (S1), dorsal and ventral premotor cortex (PMC), supplementary motor area (SMA), dorsolateral prefrontal cortex (DLPFC), posterior parietal cortex (PPC), cerebellum, and basal ganglia [15,16,17,18,19,20,21,22,23]. Within these brain regions, motor performance is associated with a decrease in the power spectrum of the alpha (~8–13 Hz) and beta frequencies (~13–30 Hz) directly before and during the execution of movement; this decrease in the power spectrum is called event-related desynchronization (ERD). On a mechanistic level, ERD in the alpha and beta frequencies in the sensorimotor areas is thought to reflect increased cortical activity, such as higher cortical excitability [24,25,26,27,28], and might be related to the degree of spike synchronization [29]. In sensorimotor cortices, movement-related beta activity is directly linked with endogenous GABA concentration, as indicated by studies showing that pharmacologically enhanced GABA activity leads to higher beta power [30,31,32]. Because decreased GABA levels were found to be associated with motor skill learning [33,34], the lower beta activity and, thus, the increased cortical excitability occurring during motor skill practice, is thought to facilitate synaptic transmission, such as LTP-like plasticity between sensorimotor networks.

In addition to their physiological effects, both alpha and beta frequency bands play a prominent role in producing motor actions (e.g., motor planning and execution) and are also suggested to be related to motor skill learning. Along these lines, alpha and beta activity show modulations associated with motor skill learning progress, but their functional roles are not well understood. Several studies have shown that higher alpha power (less alpha ERD) is generated during motor practice when motor performance increases [35,36,37,38]. These changes were thought to occur because fewer attentional and cognitive demands are required when a motor task becomes more automated. Similarly, beta oscillations also seem significant in motor learning. A stronger beta ERD during motor practice was correlated with increased motor performance [39], and was linked to superior learning gains after motor practice [37]. Furthermore, the degree of beta ERD seems to be strongly related to motor learning, as recently indicated for both healthy participants and patients with Parkinson’s disease [40]. Hence, beta ERD is assumed to be a potential biomarker of neuroplasticity during motor skill learning.

To date, few studies have investigated the effects of aCE on motor performance and learning and on brain oscillations by investigating EEG activity during motor practice and retention. Recently, Dal Maso et al. [6] investigated whether 15 min of high-intensity interval aCE (workload of 90% VO_2peak_) conducted on a stationary bicycle ergometer affected learning of a visuomotor tracking task (VMT). The high-intensity interval aCE was applied following motor practice, and the effects were compared to an inactive control group (sitting on the bicycle ergometer without exercising). Interestingly, the exercise group showed higher learning gains after a second retention interval of 24 h (after exercise/rest), suggesting that a single bout of high-intensity interval aCE facilitated sleep-related offline consolidation in the practiced VMT. In association with the behavioral results, Dal Maso et al. reported weaker ERD (less cortical activation) in bihemispheric sensorimotor areas at the beta frequency (15–29 Hz) in the exercise group compared to the inactive controls 30 min after exercise cessation, and as participants performed an isometric handgrip task with the dominant right hand (untrained task). These changes in beta power were further correlated with the improved offline consolidation following the 24 h retention interval. However, although the alpha frequency band is involved in motor processing, they found no effect of high-intensity aCE on alpha power (8–12 Hz).

In a recent study by our group, [41] Hübner et al. investigated whether a moderate aCE intervention (60% of watt_max_) affected learning of a visuomotor force-matching task (FM task) in healthy older adults (aged 65–74 years). Furthermore, they also investigated task-related changes in beta EEG power (13–30 Hz), which has been shown to be related to FM task performance before [42]. The moderate aCE was performed for 20 min on a stationary bicycle ergometer and, unlike to Dal Maso et al. [6], this took place before motor practice. The findings revealed improved motor performance in the moderate aCE group compared to an inactive control group immediately after the intervention. Improved motor performance due to the moderate aCE was associated with a steeper decrease in beta task-related power (TRPow) at the contralateral frontal site (F3), and this was also more strongly decreased during initial motor learning. Hence, due to moderate aCE, more substantial TRPow decreases in older adults’ brains seem to be linked to superior motor performance. However, the moderate aCE performed before motor practice only slightly improved the early motor memory of the FM task.

Thus, given these previous studies, in order to gain more understanding of the underlying neurophysiological processes, we aimed to investigate the effects of high-intensity aCE on motor performance and learning, as well as on the associated changes in TRPow in the alpha (α; 8–13 Hz) and beta (β; 13–30 Hz) frequency bands. Therefore, we used a similar study protocol to that used by our group previously [41], but in a group of young adults. The effects were compared to a low-intensity aCE group (active control) and an inactive control group (resting condition without exercise). We hypothesized that a single bout of high-intensity aCE performed before motor practice would lead to enhanced FM task performance immediately after aCE cessation, superior initial learning gains following motor practice (online learning), and improvements in later learning stages interspersed by phases without motor practice (offline learning). Furthermore, we assumed that enhanced motor performance and superior learning gains due to high-intensity aCE would be paralleled by modulations in oscillatory brain activity. First, we expected a progressive increase in *α*-TRPow throughout motor learning, showing a more substantial increase after high-intensity aCE associated with superior learning gains. Second, we expected a decrease in *β*-TRPow due to motor practice, which would be even steeper and associated with superior learning due to high-intensity aCE.

## 2. Materials and Methods

### 2.1. Participants

Forty-five healthy young adults aged between 18 and 34 (mean age 23.18 ± 4.01; 27 female) participated in the experiment. Participants were recruited via student mailing lists and flyers announcing the following prerequisites for study participation: (1) absence of neurological and cardiovascular diseases, (2) physically active lifestyle, and (3) right-handedness. The Ethics Committee of the Faculty of Humanities of the Saarland University (ethical approval code 4.3.13) approved the study. Participants provided informed consent regarding general study information, EEG, and the cardiovascular fitness test, and took part voluntarily. They received a personalized analysis of their cardiovascular fitness and/or credits for a university course (note that the inactive control group did not perform a cardiovascular fitness test).

Participants answered a questionnaire assessing demographic information, education level (years of education), and subjective health status (“In general, how would you say your health is?” on a 5-point Likert scale from poor to excellent). The Edinburgh Handedness Inventory [43] confirmed their right-handedness (overall score: 85.44 ± 17.21). Additionally, participants were asked about their fine motor activities during daily life [44]. The groups were equally sized, and participants were matched based on individuals’ gender, age, and cardiovascular fitness level, to a high-intensity exercise group (HEG; *n* = 15, 8 female), a low-intensity exercise group (LEG, *n* = 15, 9 female), and an inactive control group (CG; *n* = 15, 10 female) (for an overview and group comparisons, see Table 1).

### 2.2. Cardiovascular Fitness Test

Cardiovascular fitness was assessed via spiroergometry (ZAN600, nSpire Health, Oberthulba, Germany) on a stationary bicycle (Lode Corival cpet, Groningen, The Netherlands). A ramp protocol with a continuously increasing workload (15 to 25 watts over 1 min) was used to determine maximal oxygen consumption (VO_2_-max). Since it is suggested that participants reach their limits of cardiovascular performance in approximately 10–12 min [45], the protocol was adjusted according to participants’ gender and self-reported physical activity levels, defined as at least (equals the “fit” protocol) or less than (equals the “unfit” protocol) three hours per week of endurance sports, such as swimming, running, or cycling. Therefore, a particular ramp protocol was chosen for each participant: the female “unfit” protocol had a load increase of 15 watts/min, with 10 watts as the starting load; the female “fit” and male “unfit” protocols had a load increase of 20 watts/min, with 20 watts as the starting load; the male “fit” protocol had a load increase of 25 watts/min, and started at 25 watts [46]. We continuously monitored participants’ electrocardiography (ECG; recorded with a 10-lead ECG fully digital stress system; Kiss, GE Healthcare, Munich, Germany), breath-by-breath respiration, heart rate (HR), and blood pressure. An experienced sport scientist supervised all tests. Spiroergometry protocols started with a 3-minute rest period. Afterwards, participants were asked to keep their revolutions per minute (rpm) between 60 and 80, and to cycle until their volitional exhaustion. Objective exhaustion criteria were defined as a respiratory quotient (ratio of carbon dioxide and oxygen) of 1.3 for ~30 s, blood pressure above 230/115 mmHg, or an HR above 220 minus the participant’s age. Tests were finished with a 5-minute cool-down period. The values of the highest complete performance level (approximately ≥ 4–5 s) were averaged and regarded as VO_2_-max (VO_2_ in l/min).

### 2.3. Visuomotor Force-Matching Task (FM Task)

**Figure 1:**
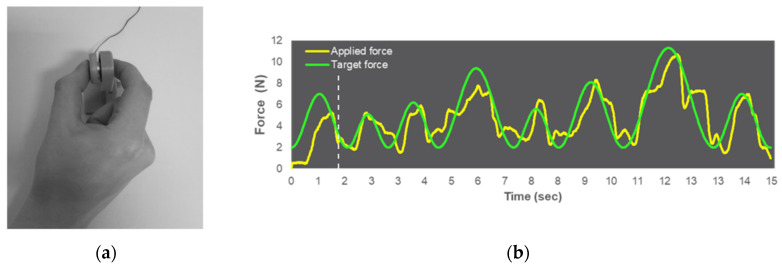
(**a**) Participant performs a precision grip on the force transducer (FT). (**b**) Example illustration of the required sine wave pattern of the FM task with the target force (green line) and applied force (yellow line). The dashed line represents the starting point of the data analysis (1.75 s after the trial’s onset), and was not visible during the experiment.

The visuomotor force-matching task (FM task) was performed with a pinch grip using the thumb and index finger of the dominant right hand. FM task performance was assessed using a force transducer (FT; FX1901 OEM sensor, Variohm Eurosensor, Heidelberg, Germany) with a 25 mm diameter sensor plate encased in a 2.6 mm diameter plastic sheath (see Figure 1). Pinch force data were recorded at a sampling rate of 120 Hz and a resolution of 0.06 N using a customized LabVIEW program (LabVIEW 2015, National Instruments Austin, TX, USA). The participants were seated in front of a table at a distance of ~60 cm from a 23.8-inch monitor (hardware resolution: 1920 × 1080 pixels). The FT was placed on a table in a comfortable position for participants’ use with their dominant right hand.

The participants’ maximum voluntary contraction (MVC) was measured by requesting them to exert as much force as possible with their thumb and index finger on the FT (3 trials of 5 s, ≥30 s rest between trials), while online visual feedback about the applied force was presented on the monitor as a yellow line contrasted to a black background. The highest value out of the 3 trials was regarded as the MVC.

In the FM task, participants were asked to match their own applied pinch force (presented on the monitor as a yellow line) to a given target force (shown on the monitor as a green line) as accurately as possible. The target force consisted of an irregular sine wave pattern of eight sine waves with the same minima (2 N) and varying maxima (5.1–11.3 N), and appeared 200 ms before the applied force from the right-hand screen margin, moving to the left [41]. The tracking velocity was identical within each sine wave, since the sine wave frequency was adapted to the varying maxima (0.35–0.78 Hz). The same sine wave pattern was performed repetitively throughout the experiment (13 blocks of 8 trials; 104 trials in total), with a single trial length of 15 s. All trials were followed by an inter-trial break of 4 s, during which a white fixation cross appeared on a black screen. The inter-trial breaks occurred in all blocks, where each block consisted of 8 trials, so the total duration for one block was 148 s. None of the participants had any experience with the task before the experiment. FM task files are available upon request to the corresponding author.

### 2.4. EEG Recording

The EEG was recorded with a 32-channel EEG device using active electrodes (actiCHamp, Brain Products, Gilching, Germany) at a sampling rate of 500 Hz. Electrodes were placed in a nonconductive EEG cap (actiCAP, EASYCAP GmbH, Woerthsee-Etterschlag, Germany) and positioned at Fp1, Fp2, F7, F3, Fz, F4, F8, FC5, FC3, FC1, FC2, FC4, FC6, T7, C3, Cz, C4, T8, CP5, CP3, CP1, CP2, CP4, CP6, P7, P3, Pz, P4, P8, O1, Oz, and O2 according to the international 10–10 EEG system. FPz served as ground, and Fz was used as the reference electrode. Active electrodes (Ag/AgCl) were filled with highly conductive electrolyte gel (SuperVisc, EASYCAP GmbH, Woerthsee-Etterschlag, Germany) to reach low impedance levels (<5 kΩ). EEG recordings were conducted during rest, as well as during FM task performance. Participants were instructed to sit comfortably but calmly on the chair for rest measurements (60 s with opened eyes), with relaxed facial muscles, both hands lying on the table, and looking at a white fixation cross presented on a black screen.

### 2.5. Acute Exercise Session

The acute exercise groups (HEG and LEG) performed an exercise session by cycling on a stationary ergometer (Lode Corival cpet, Groningen, the Netherlands). The HEG protocol was mainly based on previous studies that evaluated the effects of high-intensity cardiovascular exercise on motor performance and learning [5,7,8,10]. For detailed information about the acute exercise protocols, see Table 2. In both groups, HR was continuously monitored using a Polar A300 (Polar Electro Oy, Kempele, Finland) with an H7 heart rate sensor (Polar Electro Oy, Kempele, Finland). The inactive control group (CG) listened to an audiobook (narrative short story) for ~25 min.

### 2.6. Procedure

**Figure 2:**
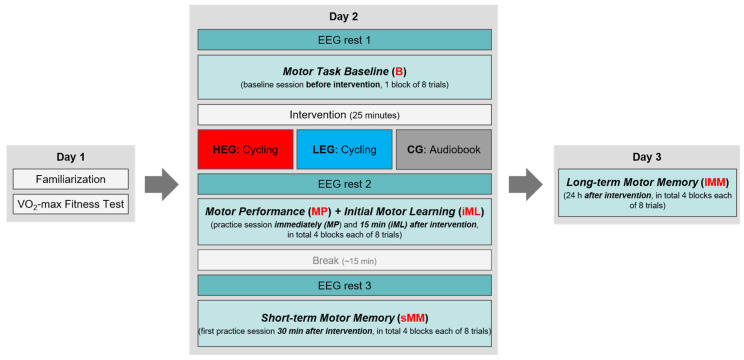
Schematic illustration of the study design: Text in bold italics represents timepoints of the FM task included for statistical analyses (each timepoint consists of 1 block of 8 trials). EEG boxes denote the timepoints of EEG measurement during rest, while EEG was also measured continuously during the FM task on day 2.

The study protocol was identical to our former study by Hübner et al. [41], aside from the acute exercise protocols. The study took place over three experimental days (see Figure 2). On day 1, participants signed the informed consent for study participation, performed the MVC test, became familiarized with the FM task, and then performed the cardiovascular fitness test. For FM task familiarization, participants were asked to try out the FT to see how it reacts to low-force pinching (modulations of the yellow line, as an indicator for their own applied force) without a target curve (1 trial, length: 10 s). Then, participants were asked to match target forces as accurately as possible—first to a constant target line at 4 N (3 trials of 5 s each), and then to a regular sine wave pattern between 1 N and 5 N with a frequency of 0.4 Hz (3 trials of 6.67 s each). This activity was only for familiarization, and was not evaluated. To ensure a complete recovery from the cardiovascular fitness test, the delay between days 1 and 2 was at least 48 h.

On day 2, first the EEG cap equipped with electrodes was mounted on the participant’s head, and the heart rate monitor was placed. Then, the electrophysiological measurements (EEG and HR) were checked to ensure that the equipment worked properly. After that, a resting EEG was conducted (rest 1) in a seated position on a chair (~2 min). Participants then performed one block of the FM task as a baseline measurement (*B-block*) while, concurrently, the EEG was assessed. Subsequently, the cardiovascular exercise (HEG and LEG) or the inactive control condition (CG) took place (during the exercise/control condition, no EEG was measured, but participants were still equipped with the EEG cap). Immediately after exercise/control cessation, a second resting EEG was assessed (rest 2) and, subsequently, participants practiced the FM task over 4 blocks broken up by short breaks of 30 s (the first block of the practice session started approximately 2–5 min after exercise/control cessation). Completion of the fourth practice block was followed by a 15-minute break, where participants stayed on the chair and talked with one of the examiners. Finally, ~30 min after the end of the exercise/control condition, resting EEG was again assessed (rest 3), and the participants performed additional 4 blocks of the FM task in a second practice session. In total, 9 blocks with in sum 72 FM task trials were conducted on day 2. Subjective fatigue of the task-performing right hand was assessed on a scale from 0 (not at all fatigued) to 10 (totally fatigued) before baseline (Fatigue 1), after the first practice session (4 blocks) immediately after the intervention (Fatigue 2), and after the second practice session (4 blocks) 30 min after the intervention (Fatigue 3; see Table 3). Similarly, HR was recorded before FM task baseline (HR 1), following exercise (HR 2), and after the first and second practice sessions (HR 3 and HR 4) (Figure 2; see Table 3 for group comparisons).

On day 3, we assessed long-term motor memory consolidation 24 h (range: 23.5–24.5 h) after the first FM practice session started on day 2. Here, participants again performed 4 blocks of the FM task (32 trials in total), and subjective hand fatigue was assessed prior to (Fatigue 4) and after (Fatigue 5) the practice sessions. The order of tasks per testing day did not vary between participants.

### 2.7. Data Analysis

#### 2.7.1. FM Task Data

FM task performance was analyzed using MATLAB R2019b software (MathWorks, Inc., Natick, MA, USA). The first sine wave (first 211 data points; approximately 1.75 s) of each trial was cut off to avoid variation due to differences in ramp time. Fine motor control performance was parameterized by the root-mean-square error (RMSE), which captures the deviations of the applied force from the target force in every trial. A mean of 8 trials was calculated for each FM task block. The statistical analysis was then focused on the same 5 timepoints as in the previous study by Hübner et al. [41], aimed at assessing changes in FM task performance (mean RMSE) over time (see Figure 2): (1) *B-block* (the first block, which served as a baseline measurement before intervention/control; mean of 8 trials), (2) *MP-block* (the motor performance in the first block immediately after intervention; mean of 8 trials), (3) *iML-block* (the fourth block, immediately after intervention, defined as initial motor learning; mean of 8 trials), (4) *sMM-block* (the first retention block, defined as short-term motor memory, performed 30 min after intervention and 15 min after the last practice block; mean of 8 trials), and (5) *lMM-block* (the second retention block, defined as long-term motor memory, performed 24 h after the intervention; mean of 8 trials).

#### 2.7.2. EEG Data

The raw EEG data were preprocessed offline using BrainVision Analyzer software (Version 2.1, Brain Products GmbH, Gilching, Germany). All preprocessing steps were identical for EEG recordings during rest and FM task performance. The data were filtered using a phase-shift-free Butterworth infinite impulse response (IIR) filter. The low cutoff was set at 1 Hz and the high cutoff at 70 Hz, with a slope of 48 dB/Oct. To reduce line noise, a notch filter at 50 Hz was applied. Raw data were inspected according to the following criteria: gradient with maximally allowed voltage steps of 25 µV, lowest allowed activity of 0.5 µV. Ocular artifacts were removed via semiautomatic ocular correction using an extended-infomax independent component analysis (ICA), with the Fp1 electrode detecting both vertical and horizontal ocular movements, as confirmed via visual inspection. For analysis of EEG activity during FM task performance, the data were cut into segments reaching from the beginning of the first trial to the end of each session’s last trial on day 2. Accordingly, we removed the inter-trial breaks and the first 1.75 s of each FM task trial (corresponding to each trial’s first sine wave curve; see Section 2.7.1 above). For EEG analysis during rest, the data were cut into 20-second segments (second 5 to second 25 were used for analysis). Continuous data were further separated into segments of 2 s (overlapping segments of 150 ms), resulting in 7 segments per FM task trial and 10 segments per EEG rest. Upon visual inspection, trials containing bad segments with obvious remaining artifacts were excluded.

Following preprocessing, a fast Fourier transform (FFT) algorithm was applied (output was set to power, measured in µV^2^) using a maximum resolution (0.488 Hz), a full spectrum, and a Hanning window with a window length of 10% for each individual FM task trial and each EEG rest measurement. The 2-second-long segments were zero-padded to a length of 2048 ms. Afterwards, power spectra for the alpha (8–13 Hz) and beta (13–30 Hz) frequency bands were calculated for each trial of the FM task and each EEG rest measure, separately, for the following six electrodes of interest (EOIs): three electrodes allocated over the frontal (F3), central (C3), and centro-parietal (CP3) positions of the left (contralateral) hemisphere, and three electrodes positioned on the frontal (F4), central (C4), and centro-parietal (CP4) sites of the right (ipsilateral) hemisphere. The selected EOIs were presumed to cover bilateral brain areas (e.g., C3 overlying the left M1) involved in motor control of precision grip [47,48], as required during performance of the FM task, which has also been shown in former EEG studies using the similiar task [41,42]. Subsequently, a mean power value per electrode was calculated for each FM task trial (7 segments) and averaged separately for each block (averaged power over the 8 trials (56 segments) within *B-block*, *MP-block*, *iML-block*, and *sMM-block*) and each EEG rest measure (i.e., rest 2). Finally, task-related power (TRPow) of the alpha (*α*-TRPow) and beta (*β*-TRPow) frequency bands for each EOI was calculated using the mean power values of each FM task block and of each EEG rest measure before the required FM task block (i.e., rest 1 to rest 3), converted into decibels (dB; [49]):

$$\mathrm{TRPow}\;\left(\mathrm{dB}\right)=10*\log10\;\left(\frac{\mathrm{task}}{\mathrm{rest}}\right)$$

Accordingly, *α*-TRPow and *β*-TRPow of each EOI during the FM task blocks were calculated as follows, and expressed in dB:

$${\mathrm{TRPow}}_{B-block}=10*\log10\;\left(\frac{{\mathrm{task}}_{B-block}}{\mathrm{rest}\;1}\right)$$

$${\mathrm{TRPow}}_{MP-block}=10*\log10\;\left(\frac{{\mathrm{task}}_{MP-block}}{\mathrm{rest}\;2}\right)$$

$${\mathrm{TRPow}}_{iML-block}=10*\log10\left(\frac{{\mathrm{task}}_{iML-block}}{\mathrm{rest}\;2}\right)$$

$${\mathrm{TRPow}}_{sMM-block}=10*\log10\left(\frac{{\mathrm{task}}_{sMM-block}}{\mathrm{rest}\;3}\right)$$

TRPow changes in the alpha and beta frequency bands are associated with changes in cortical activation due to task performance [50]. Negative values in TRPow indicate increased cortical activity due to task-related desynchronization (e.g., ERD), reducing EEG power compared to oscillatory activity at rest. Conversely, positive TRPow values indicate decreased cortical activity, associated with stronger oscillatory synchronization (e.g., event-related synchronization; ERS), eliciting higher EEG power [25,51].

### 2.8. Statistical Analyses

For statistical analyses, we used a hybrid approach of classical frequentist inference and Bayesian equivalents for hypothesis testing [52,53], in order to quantify the evidence on whether high-intensity aCE enhances motor performance and learning. The same approach was used to quantify the evidence on whether modulations in *α*-TRPow and *β*-TRPow paralleled motor performance and learning. Analyses and graphics were made using IBM SPSS 26 (IBM Corporation, Armonk, NY, USA), JASP (version 0.14, JASP Team, 2020), and R (version 4.0.3).

To investigate the intervention’s effects on motor skill learning of the FM task, we conducted a (Bayesian) 5 × 3 repeated-measures ANOVA (RM-ANOVA) containing the factors TIME (*B-block*, *MP-block*, *iML-block*, *sMM-block*, *lMM-block*)—as a five-level within-subject factor—and GROUP (HEG, LEG, CG), as a three-level between-subject factor.

For EEG data, all statistical analyses were conducted identically for *α*-TRPow and *β*-TRPow, separately for each EOI (F3, F4, C3, C4, CP3, and CP4), in order to provide a differentiated view on *α*-TRPow and *β*-TRPow modulations associated with the intervention on day 2. Hence, we conducted a (Bayesian) 4 × 3 RM-ANOVA with the four-level within-subject factor TIME (*B-block*, *MP-block*, *iML-block*, *sMM-block*) and the three-level between-subject factor GROUP (HEG, LEG, CG).

In the frequentist statistics, significance was set at *p* < 0.05, and the nominal alpha level was corrected for multiple comparisons using Bonferroni adjustment (α = 1 − (1 − α)^1/m^) for the separate RM-ANOVAs. Outliers were marked as values below the first quartile (−1.5 × interquartile range (IQR)) or above the third quartile (+1.5 × IQR), and were not excluded from the analysis. Levene’s test was used to test for equality of variances, Mauchly’s test assessed the assumption of sphericity, and Greenhouse–Geisser-corrected *p*-values were reported in case of violation. Normal distribution was checked via Shapiro–Wilk tests and visual inspection of Q–Q plots. Bonferroni-corrected simple main effect analyses and pairwise comparisons were used to disentangle significant main effects and interactions. Follow-up analyses at the group and time levels were conducted to answer our a priori research questions. To facilitate cumulative science [54], ANOVA effect sizes (ESs) were reported as partial eta-squared (η^2^_p_) and generalized eta-squared (η^2^_G_), with benchmarks of >0.01 indicating a small effect, >0.06 a medium effect, and >0.14 a large effect [55]. ESs for *t*-tests were expressed as Cohen’s d (*d*), with values of >0.02 indicating a small effect, >0.05 a medium effect, and >0.08 a large effect [55].

For Bayesian ANOVAs, default priors (r scale fixed effects = 0.5 and r scale random effects = 1) were used, and effects were reported as Bayes factor inclusion (BF_incl_) for matched models. As such, the BF_incl_ provides information for the inclusion of a certain effect (e.g., interaction), indicated as the ratio between the likelihood of the data supporting the model with the effect versus the model stripped of that effect [52]. The resulting value of the BF_incl_ expresses the change from prior to posterior inclusion odds, and describes the extent to which the data support inclusion of the effect(s) under consideration [52,56]. Similar to the Bayes factor (e.g., BF_10_), the BF_incl_ represents a continuous measure to quantify the evidence for or against an effect. Reference values are provided to generally categorize the strength of evidence, where values of BF < 0.33 reveal evidence for H0; BF = 0.33–3 shows no evidence in favor of or against an effect (insensitivity of data), and BF > 3 indicates evidence for H1 [57] based on an earlier categorization scheme by Jeffreys [58]. In sum, the Bayes factor provides information about whether there is evidence of absence, or the absence of evidence, for the effect under investigation. The results of Bayesian post hoc comparisons were reported as BF_10_^U^ (the U indicates uncorrected) and as posterior odd, adjusted for multiple comparisons [59].

## 3. Results

### 3.1. Behavioral Data—FM Task

**Figure 3:**
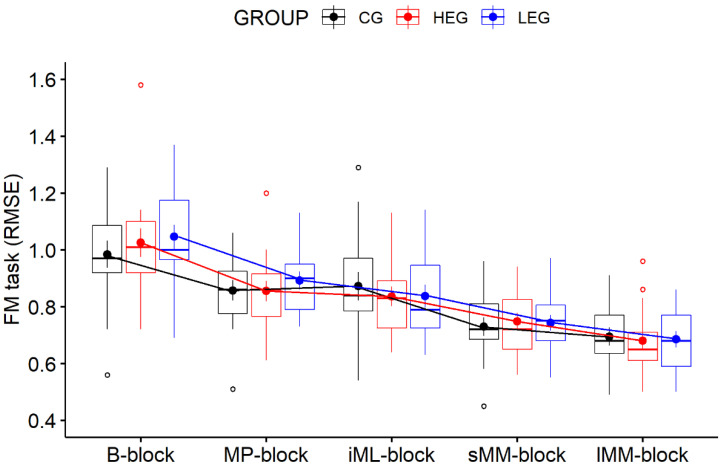
Development of FM task performance over the duration of the experiment (*B-block:* baseline; *MP-block:* motor performance; *iML-block:* initial motor learning; *sMM-block:* short-term motor memory; *lMM-block:* long-term motor memory), as indicated by boxplots containing RMSE means and (±) SEM per group (HEG, LEG, and CG). Outliers were marked as values below the first quartile (–1.5 × IQR) or above the third quartile (+1.5 × IQR) (see SupplementaryTable S1 for mean values and SD of RMSE).

As shown in Figure 3, all three experimental groups improved their FM task performance (decreased RMSE) over the duration of the experiment. The 5 × 3 RM-ANOVA revealed evidence of an overall improvement in FM task performance over time, as indicated by a main effect of TIME (*F*(4, 168) = 120.31, *p* < 0.001, η^2^_p_ = 0.741, η^2^_G_ = 0.396, BF*_incl_* = 5.673 × 10^45^). No main effect of GROUP (*F*(2, 42) = 0.32, *p* = 0.97, η^2^_p_ = 0.002, η^2^_G_ = 0.001, BF*_incl_* = 0.292) nor of a TIME × GROUP interaction (*F*(8, 168) = 0.98, *p* = 0.45, η^2^_p_ = 0.045, η^2^_G_ = 0.011, BF*_incl_* = 0.085) emerged, revealing that changes in performance over time were not affected by groups. Follow-up analyses at the group level based on our a priori research questions revealed that each group improved in FM task performance from the *B-block* to the *MP-block* (all *p* < 0.05; all BF_10_ > 3) and from the *B-block* to the *iML-block* (all *p* < 0.001; all BF_10_ > 3). These analyses show that there was no effect of aCE on immediate motor performance after exercise cessation, nor on initial motor learning. A comparable picture emerged for our proposed effects on later learning stages, since each group improved from the *B-block* to the *sMM-block* and from the *B-block* to the *lMM-block* (all *p* < 0.001; all BF_10_ > 3), revealing that later learning stages were also not affected by aCE. Interestingly, there was no improvement from the *MP-block* to the *iML-block* in any group (all *p* > 0.05; all BF_10_ < 0.33), indicating that a performance plateau had been reached during practice. Even more interestingly, only the CG did not further improve in the FM task performance from the *sMM-block* to the *lMM-block* (*p* = 0.774, *d* = 0.378, BF10 = 0.635) with a retention of 24 h in between, suggesting that exercise may have slightly facilitated motor memory consolidation (offline learning) independent of intensity. However, no group differences emerged at any timepoint (all *p* > 0.05; all BF_10_ < 0.33).

### 3.2. EEG Data—Task-Related Power

Results for *α*-TRPow and *β*-TRPow are presented separately; see Table 4 for a comprehensive overview of all statistical results of the statistical analyses described in Section 2.8.

#### 3.2.1. Changes in TRPow in the Alpha Frequency Band (*α*-TRPow)

**Figure 4:**
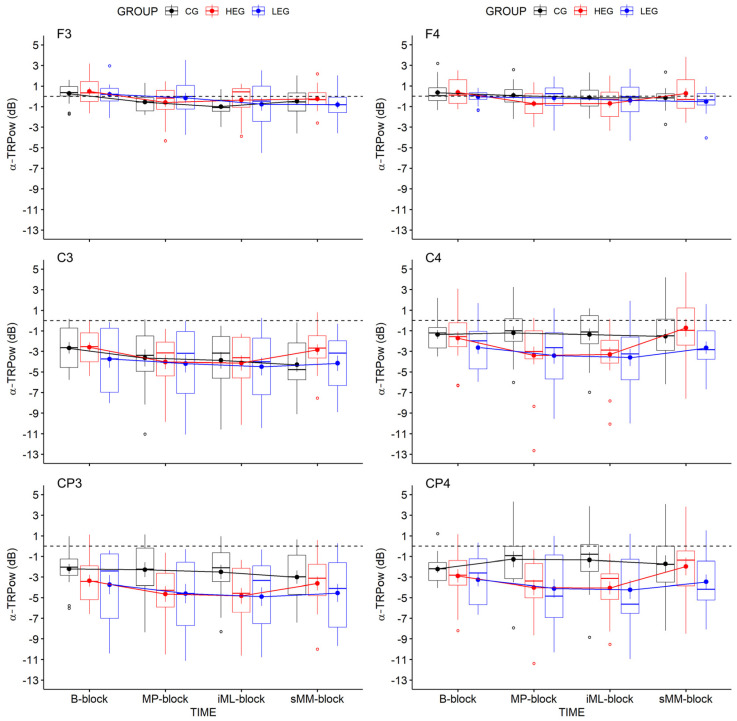
Illustration of changes in *α*-TRPow over the 4 measurement blocks included in the statistical analyses: Boxplots shown also contain RMSE means and (±) SEM per group (HEG, LEG, and CG). Negative values indicate decreased TRPow, representing increased cortical activation. Outliers are marked as values below the first quartile (–1.5 × IQR) or above the third quartile (+1.5 × IQR) (see Supplementary Table S2 for mean values and SD of *α*-TRPow).

Effects of aCE on changes in *α*-TRPow were analyzed for key electrodes (F3, C3, CP3, F4, C4, CP4), for each of which we used a 3 × 4 (Bayesian) repeated-measures ANOVA (adjusted α = 0.008; see Table 4 and Figure 4). The results revealed evidence of a decrease in *α*-TRPow over the frontal and central contralateral regions, as shown by a main effect of TIME for electrodes F3 (*F*(3, 126) = 6.05, *p* < 0.004, η^2^_p_ = 0.126, η^2^_G_ = 0.068, BF*_incl_* = 45.499) and C3 (*F*(3, 126) = 5.70, *p* = 0.006, η^2^_p_ = 0.119, η^2^_G_ = 0.025, BF*_incl_* = 20.799), while only minor evidence for a decrease at CP3 emerged (*F*(3, 126) = 3.89, *p* = 0.022, η^2^_p_ = 0.085, η^2^_G_ = 0.016, BF*_incl_* = 2.629), which was not even statistically significant. The evidence for the ipsilateral electrode C4 remained insensitive (*F*(3, 126) = 3.52, *p* = 0.043, η^2^_p_ = 0.077, η^2^_G_ = 0.034, BF*_incl_* = 1.624), and no evidence for the electrodes F4 and CP4 was found. The changes in *α*-TRPow were not affected by group, since no evidence for TIME × GROUP interactions or main effects of GROUP occurred (see Table 4).

Follow-up analyses of TIME at the group level showed group-specific effects only for the HEG over C3. *α*-TRPow at C3 decreased from the *B-block* to the *MP-block* (*p* = 0.046, *d* = 0.739, BF_10_ = 4.639) and from the *B-block* to the *iML-block* (*p* = 0.025, *d* = 0.739, BF_10_ = 4.631). These findings suggest that high-intensity aCE transiently modulated the *α* activity within the contralateral M1 (C3) during immediate motor performance, as well as initial motor learning, whereas no evidence for effects on later stages emerged.

#### 3.2.2. Changes in TRPow in the Beta Frequency Band (β-TRPow)

**Figure 5:**
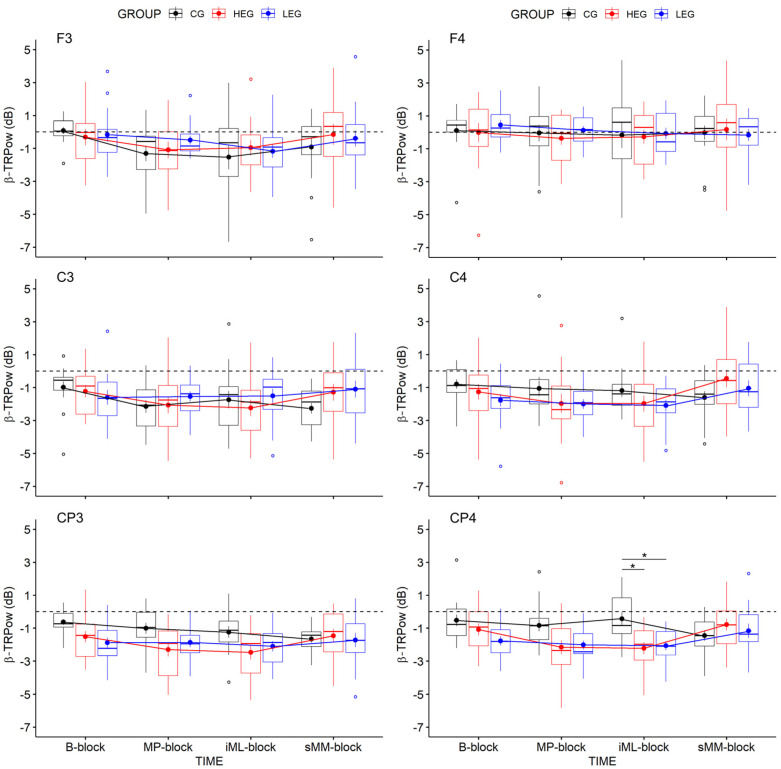
Illustration of changes in *β*-TRPow over the 4 measurement blocks included in the statistical analyses: Boxplots shown contain RMSE means and (±) SEM per group (HEG, LEG, and CG). Negative values indicate decreased TRPow, representing increased cortical activation. Outliers are marked as values below the first quartile (–1.5 × IQR) or above the third quartile (+1.5 × IQR). *: *p* < 0.05 (see Supplementary Table S3= for mean values and SD of *β*-TRPow).

**Figure 6:**
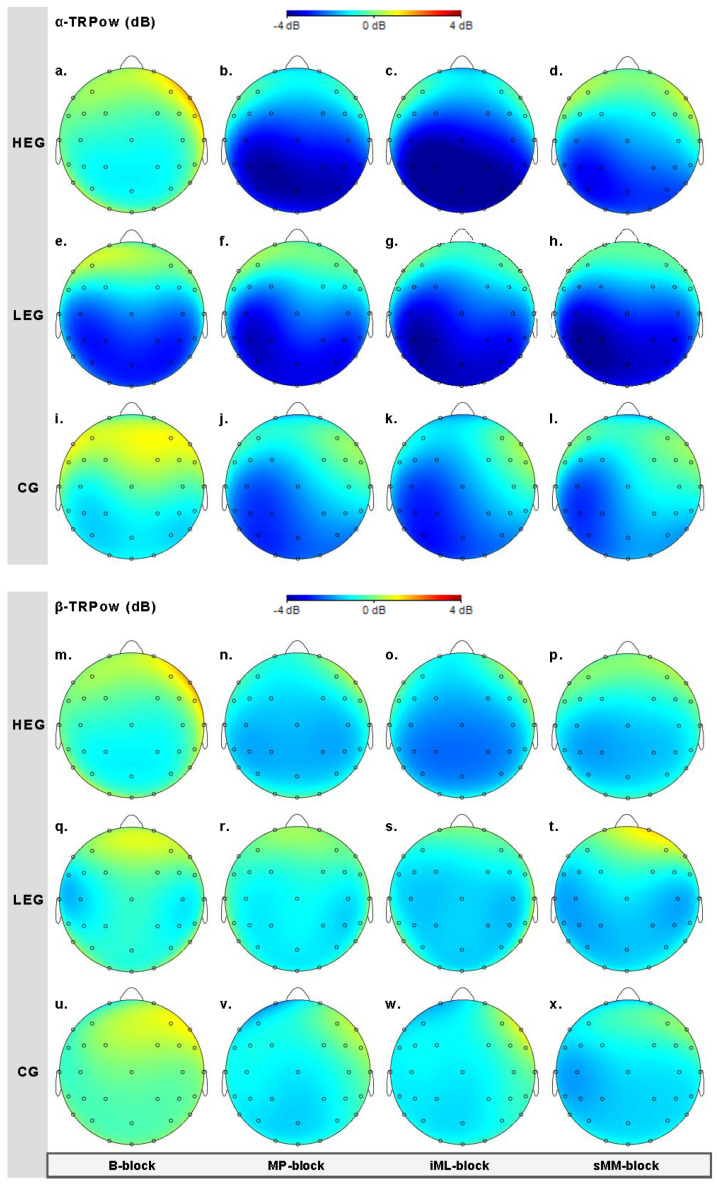
EEG scalp maps of *α*- and *β*-TRPow (dB) for the high-intensity exercise group (HEG), low-intensity exercise group (LEG), and control group (CG) over the 4 measurement blocks included in the statistical analyses (*B-block:* baseline; *MP-block:* motor performance; *iML-block:* initial motor learning; *sMM-block:* short-term motor memory).

Figure 5 illustrates the effects of aCE on *β*-TRPow. Analyses revealed evidence in favor of a TIME x GROUP interaction (adjusted α = 0.008) at the ipsilateral electrode CP4 (*F*(6, 126) = 3.90, *p* = 0.005, η^2^_p_ = 0.157, η^2^_G_ = 0.088, BF*_incl_* = 31.463). Subsequent Bonferroni-adjusted simple main effect analyses and post hoc comparisons decomposed the TIME x GROUP interaction, revealing evidence of an effect of GROUP at the *iML-block* (*F*(3, 42) = 7.96, *p* = 0.004, η^2^ = 0.275, η^2^_G_ = 0.275, BF*_incl_* = 30.419), and post hoc pairwise comparisons between groups revealed a stronger *β*-TRPow decrease in the HEG versus the CG (*p* = 0.002, *d* = 1.210, BF_10_^U^ = 14.621, posterior o = 8.589) and in the LEG versus the CG (*p* = 0.006, *d* = 1.219 BF_10_^U^ = 15.334, posterior o = 9.007). These results suggest that ipsilateral activity in the cento-parietal region seems to be affected independent of exercise intensity, and occurs around 15 min after exercise cessation, as can also be seen in the EEG scale maps (Figure 6o,s,w).

The results suggest that the centro-parietal cortex—especially the ipsilateral side—seems to be affected by aCE, with more substantial effects from high-intensity aCE.

## 4. Discussion

This study’s purpose was to quantify evidence on whether a single bout of high-intensity cardiovascular exercise (aCE) has an enhancing effect on motor skill performance immediately after exercise intervention, or on motor learning stages (initial, short term, and long term) following motor practice. As we considered that the effects on the behavioral level are paralleled by modulations in task-related EEG power, we tested the hypothesis that the beneficial effects of high-intensity aCE on motor performance and on learning a visuomotor force-matching task (FM task) would be associated with modulations in *α*-TRPow and *β*-TRPow in the sensorimotor cortex in healthy young adults. The behavioral results revealed no evidence of an effect of high-intensity aCE—neither on immediate motor performance nor on motor skill learning—compared to a low-intensity exercise group or to an inactive control group. The findings in EEG activity showed a contralateral decrease in *α*-TRPow over time, independent of the group, while slight differences between groups emerged—such as stronger decreases in the HEG in *α*-TRPow over the contralateral motor cortex (C3) immediately after exercise, and during the first learning block, around 15 min after exercise cessation. Similarly, *β*-TRPow showed a stronger decrease in the ipsilateral sensorimotor cortex (CP4) in both exercise conditions (HEG and LEG) compared to the CG, indicating an exercise-induced modulation of the centro-parietal region, independent of exercise intensity.

### 4.1. Effects on Motor Performance and Learning

Although studies have shown beneficial effects of high-intensity aCE on motor skill learning when applied before motor practice [5,7,8], we found no evidence of such an enhancement in our experiment. While all participants successfully learned the FM task, as indicated by improved performance over time, high-intensity aCE seems to have no additional beneficial effects—even with improvements after two phases without practice (after 30 min in the *sMM-block* and after ~24 h in the *lML-block*) (see Figure 3). The results add to the heterogeneous findings reported by earlier studies, which also showed that the aCE does not affect motor performance [8,60,61], initial motor learning [8,10,60,62], or motor memory consolidation [60,61,62] for tasks performed directly after aCE. Interestingly, a performance plateau spanning from the *MP-block* to the *iML-block* emerged, indicating no improvements due to motor practice (4 blocks and 32 trials in total); this was interpreted as the absence of any online learning gains, independent of the intervention. However, even after a short phase without practice, offline learning gains were shown by further FM task improvements from the *iML-block* to the *sMM-block* in all groups. Even more interestingly, further improvements were found after a retention interval of 24 h, but only in the two exercise groups and not in the CG. Hence, it seems that exercise may facilitate offline learning over a longer time period without practice. Since others indicated superior offline learning gains occurring around one week after an aCE intervention [7,8], it might be possible that such delayed effects could also have been induced by our exercise protocol, but this needs to be investigated in the future.

### 4.2. Effects on Task-Related Power during Motor Performance and Learning

EEG analyses mainly showed decreases in *α*- and *β*-TRPow during FM task performance, which was more pronounced in the bilateral central and centro-parietal regions (see Figure 6). These findings are consistent with earlier studies using visuomotor force-matching tasks requiring fine motor control [42], as also shown by fMRI [47,48]. Over the duration of the experiment, *α*-TRPow progressively decreased over the contralateral frontal, central, and centro-parietal regions (Figure 6a–l). While these modulations were not associated with the intervention, follow-up analyses revealed that *α*-TRPow decreased only in the HEG from baseline (*B-block*) to immediate motor performance after aCE cessation (*MP-block*), and from baseline to early motor learning (*iML-block*). This effect was observed over central electrode C3 covering the M1 hand area. However, these suggested modulations in *α*-TRPow due to high-intensity exercise did not affect immediate motor performance or initial motor learning gains.

Moreover, the progressive decrease in *α*-TRPow is contrary to the findings of previous studies that showed a progressive increase in *α*-TRPow (higher *α*-power) due to motor learning [35,36,37,38]. As activity in the alpha frequency band has been linked to attentional information processing and automatic motor control [38,63,64,65], higher *α*-power is thought to be an indicator of less or stable motor–cognitive demand and attentional control after a motor task has been learned; however, this assumption could not be confirmed in our study, perhaps because of the ongoing performance improvements we saw, as indicated by increases in accuracy; this may mean that participants still needed a certain amount of attentional processing and sensorimotor control.

In contrast, *β*-TRPow did not change over time, indicating a constant level of cortical activation during motor performance and learning (Figure 6m–x). Here, a slight decrease in *β*-TRPow emerged, but was not associated with the groups. These findings are comparable to those of previous studies reporting no changes in *β*-ERD over motor practice [39,66,67], but they contrast with those of other studies reporting changes in *β*-ERD after motor practice [37,39,40], such that modulation in *β*-ERD may reflect functional sensorimotor reorganization associated with motor learning [37,40,68]. However, our findings do not confirm specific modulations, such as stronger decreases in *β*-TRPow parallel to improved motor performance and motor learning.

Notably, exercise-induced effects on *β*-TRPow emerged over the ipsilateral centro-parietal cortex at electrode CP4 (see Figure 6m–o,q–s,u–w). Here, *β*-TRPow showed a stronger decrease in both exercise groups (HEG and LEG) relative to the CG during the *iML-block,* which was performed approximately 15 min after the intervention (see Figure 6o,s,w). The results indicate a higher cortical activation in the ipsilateral somatosensory cortex, independent of exercise intensity. Hence, high-intensity exercise may trigger cortical activity in the ipsilateral centro-parietal cortex immediately after exercise cessation, and also with a kind of delayed effect; however, neither are associated with immediate motor performance or initial motor learning gains.

Although changes in ipsilateral activity during unimanual movements appear contradictory at first glance, recent work supports the notion that ipsilateral activity plays an important role even in unimanual (contralateral) motor control (for reviews, see [69,70]). Several studies across different methodologies have revealed consistent bilateral brain activity in unimanual movements, thus raising the question of whether unimanual motor actions have a bilateral neural representation [42,69,71]. Hence, it is possible that ipsilateral motor activity during unimanual movements could be associated with several functions, such as balancing interhemispheric inhibition between the dominant and non-dominant sides, or active motor planning and execution. These considered functions may operate within and/or between both cortical hemispheres due to pathways connecting several motor and non-motor regions in the frontal and parietal cortices within one hemisphere, or even between both hemispheres. Corresponding areas in both hemispheres are monosynaptically connected via the corpus callosum; therefore, a change in activity on one side can directly affect activity on the other side.

A recent study using transcranial direct current stimulation (tDCS) and fMRI suggests a more cooperative than competing role between the two hemispheres during unimanual motor learning [72]; these results revealed that bihemispheric tDCS increased motor learning irrespective of whether the anode, as the active electrode, was placed over the contralateral or the ipsilateral M1, and it was more effective than unihemispheric tDCS. The beneficial effects on motor learning were accompanied by increased bilateral activation in both hemispheres [72]. Although our EEG data are consistent with such previous findings, and show bilateral activations—as indicated by decreased α- and *β*-TRPow in both hemispheres during the FM task (Figure 4 and Figure 5)—only the ipsilateral activity was affected by the exercise intervention, irrespective of exercise intensity (Figure 6).

Since the more substantial ipsilateral decrease in *β*-TRPow we observed due to exercise (HEG and LEG) seems not to affect FM task performance, its functional relevance remains unclear. Interestingly, the difference in *β*-TRPow occurred in the *iML-block*, which was the fourth practice block after the intervention, and where no improvements in FM task performance occurred compared to the *MP-block* (the first practice block after the intervention). Hence, the stronger ipsilateral activity in both exercise groups may emerge as a compensatory mechanism, such as by changing the balance of interhemispheric inhibition*,* to stabilize FM task performance. This is supported by results on bimanual tasks in which one hand generates a constant force and the other hand generates a varying force comparable to the FM task [71]. This finding contributes to the suggested active role of the ipsilateral hemisphere during unimanual movements [70]. Since such an effect did not emerge in the CG, exercise may have affected the ipsilateral activity at the end of online learning after practice, independent of intensity.

Another perspective arises from transcranial magnetic simulation (TMS) studies that report an increase in cortical excitability 10–15 min after moderate- [73] or high-intensity CE [74] performed on a cycling ergometer. As the *iML-block* took place approximately 15 min after the aCE, the stronger ERD—indicated by a stronger decrease in *β*-TRPow—may reflect the increased cortical excitability induced by exercise, and this may account for the discrepancy in cortical activity between both exercise groups compared to the inactive CG, whereas no behavioral effects emerged. Our results suggest that exercise affects cortical excitability even at lower intensities, as has been observed previously using TMS [75]. In turn, our findings point to the importance of having an inactive control group, as changes in cortical excitability seem to be evoked even with lower exercise intensity levels. As such, it might be plausible that the more substantial decrease in *β*-TRPow was mainly induced by exercise, and not functionally relevant for the FM task. However, we did not directly assess cortical excitability, limiting a direct transfer of previous results to the observed ipsilateral EEG activity in our study.

The well-comparable study by Hübner et al. [41] indicated a relationship between decreased *β*-TRPow over the left frontal cortex (F3) and improved motor performance, as well as a trend for initial motor learning after moderate aCE in older healthy adults, which may serve as a compensatory neural mechanism for age-related decline in fine motor control. However, Dal Maso et al. [6] reported associations between lower *β*-ERD in the contralateral and ipsilateral motor cortices and motor skill learning in healthy young adults, reflected by an increased motor skill consolidation (24 h) in participants who conducted high-intensity aCE after motor practice. Even though our study could not find such enhancing effects at the behavioral level, it seems that beta activity might be a putative biomarker for the effects of exercise, while its functional role in motor learning remains to be elucidated. Hence, our observed decrease in *β*-TRPow around 15 min after exercise cessation might in fact be due to exercise-induced changes in cortical excitability.

Moreover, the Dal Maso study showed no effects of exercise on alpha oscillations, while we observed known modulations in the alpha frequency band over the course of motor practice, independent of intervention, supporting the functional role of alpha activity in the context of motor learning, as previously described.

Although the neural mechanisms by which exercise affects motor performance are still under investigation, the timepoint of aCE in relation to motor practice appears to be a crucial factor, as outlined previously [2,4,7,76]. Although some similarities exist between our study and the study by Dal Maso et al., in our study the aCE took place before motor practice, whereas it was applied afterwards by Dal Maso et al. This discrepancy in aCE timing in relation to motor practice may contribute to our findings and underline the importance of evaluating the proper aCE timing, which seems to still be debatable, since studies directly addressing this issue have demonstrated heterogeneous results when comparing the effects of pre-practice aCE on motor learning with those of post-practice aCE [4,7,77,78]. However, the timing of aCE before motor practice emerged as being beneficial for fine motor performance in healthy older adults [41]. Hence, one may speculate as to whether the proper timing of aCE application varies between age groups; this interpretation must be addressed in future studies, since little is known about the effects of acute exercise on motor learning in older adults [79]. Moreover, exercise intensity and timing of exercise may interact, whereby moderate-intensity exercise applied before motor practice may affect early stages of motor skill learning, whereas post-practice exercise of a high intensity may improve later stages of the learning process, such as motor consolidation, as has been discussed previously [4].

Nevertheless, the effects of aCE on motor skill learning are still not well understood in general, and more studies that systematically investigate electrophysiological correlates of such effects are required.

### 4.3. Limitations

Our study provides further insights into the effects of aCE on cortical activity accompanying motor performance and learning, but several limitations need to be stated.

We did not assess EEG during the second retention task 24 h after the exercise intervention. Although we found no behavioral effects on long-term motor memory, future studies should use EEG to evaluate changes in movement-related brain activity, which may account for superior retention due to aCE. Another point is the chosen time interval for retention tests (30 min and 24 h after exercise); since other studies found effects on short-term consolidation a couple of hours after exercise, we cannot rule out the possibility that our aCE intervention affected motor skill learning after a time period beyond 30 min after exercise, since after a prolonged time period of 24 h that effect may have washed out, or might only be seen after longer retention times of a couple of days [7,8]. Concerning the used FM task, we did not evaluate whether the sine wave pattern was learned or whether the fine motor control for producing the pinch force was facilitated by practice (e.g., improved somatosensibility). This could be addressed in the future by using a different but comparable pinch force task, e.g., using a different or random sine wave pattern for the FM task, or evaluating somatosensibility at the fingertips. Similarly, transfer effects could be assessed by using different FM task parameters over the time course of motor learning. Furthermore, the investigator who analyzed the data was not blinded to group assignment, which might represent a further study limitation. These aspects are limiting factors of our study, and should be addressed in future studies.

## 5. Conclusions

The present study investigated the effects of high-intensity aCE on motor performance and learning of a well-elaborated visuomotor force-matching task in young adults, compared to a low-exercise aCE group and an inactive control condition. The main findings indicated that aCE (high or low intensity) did not improve motor performance immediately after exercise, nor did it improve motor skill learning, as assessed in two retention tasks, 30 min and 24 h after exercise cessation. Therefore, we can conclude that the aCE protocols we used did not facilitate neuroplastic processes as proposed. The EEG data—more precisely, ipsilateral *β*-TRPow—showed a more substantial decrease during motor practice approximately 15 min after exercise cessation. This effect was observed in both exercise groups (high- and low-intensity groups), and we can conclude that it is independent of exercise intensity, but might be time-dependent. The drop in *β*-TRPow might have been related only to exercise-induced alterations in cortical excitability, because it did not affect motor performance. However, it might have also been evoked due to interhemispheric interactions required for motor control of the FM task. Future studies are necessary in order to investigate the cortical mechanisms of exercise-induced effects on motor skill learning, and should use comparable methodological and analytical approaches of exercise parameters and motor tasks to facilitate reproducibility, and replications are required. Furthermore, it might also be worthwhile to investigate other EEG parameters and frequency bands, such as the gamma frequency, which seems to play a prominent role in motor activity [80], or the use of sophisticated machine learning approaches to analyze EEG activity beyond the traditional frequency categories [81].

## Figures and Tables

**Table 1:** Group characteristics and results of one-way ANOVAs for group comparisons.

	HEG (*n* = 15, 8 ♀)		LEG (*n* = 15, 9 ♀)		CG (*n* = 15, 10 ♀)		*F*-Statistics			Bayes
	*M*	*SD*	*M*	*SD*	*M*	*SD*	*F*	*p*	*η^2^*	BF_incl_
Age	23.60	3.96	23.80	4.96	22.13	2.90	0.77	0.472	0.03	0.280
Education	16.01	3.65	15.83	3.26	15.48	2.00	0.13	0.883	0.01	0.179
Subj. health	4.13	1.06	4.20	0.68	4.47	0.64	0.70	0.501	0.03	0.268
Subj. hand usage	17.07	2.74	17.20	2.54	19.60	3.18	3.81	0.030	0.15	2.184
MVC (N)	64.70	16.39	61.51	20.39	64.35	16.35	0.14	0.866	0.01	0.375
Max. watt	225.20	53.39	207.73	83.46	NA	NA	0.01	0.926	<0.01	0.411
VO_2_-max	2.73	0.75	2.84	0.92	NA	NA	0.13	0.726	0.01	0.440

Notes—**♀**: female; Age: age in years; Education: years of education; Subj. health: self-rated health status on a Likert scale from 1 (poor) to 5 (excellent); Subj. hand: self-reported hand use (sum score of 9 items, 5-point scale); MVC: maximum voluntary contraction of index finger and thumb; (N): maximal value out of 3 trials, in newtons; Max. watt: maximum watts generated during cardiovascular fitness test; VO_2_-max: VO_2_-max performed during cardiovascular fitness test in l/min (not assessed in the CG); BF_incl:_ Bayes factor to quantify the evidence of ANOVA effects.

**Table 2:** Acute exercise protocols for the high-intensity exercise group (HEG) and low-intensity exercise group (LEG), and details (mean and *SD*) of heart rate (HR) and physical load during both exercise conditions.

	HEG	LEG
Warm-up	5 min at 40% max watt	25 min at 20% max watt
Exercise	3 × 3 min at 90% max watt interspersed with 2 × 2 min at 60% max watt	
Cool-down	5 min at 20% max watt	
HR (bpm)	90%: 175.91 ± 12.97 60%: 168.65 ± 15.96	20%: 104.20 ± 10.22
Load (watt)	90%: 208.32 ± 47.63 60%: 138.88 ± 31.75	20%: 44.76 ± 11.95

**Table 3:** Group comparisons (independent *t*-tests and one-way ANOVA) of heart rate and subjective hand fatigue ratings.

	HEG		LEG		CG		Statistics				
	*M*	*SD*	*M*	*SD*	*M*	*SD*	*t*	*df*	*p*	*d*	BF_10_
HR 1	82.00	15.03	78.07	11.59	NA	NA	0.80	28	0.429	0.293	0.440
HR 2	102.53	15.70	77.00	11.51	NA	NA	5.08	28	<0.001	1.855	760.419
HR 3	92.47	14.92	73.40	8.81	NA	NA	4.26	28	<0.001	1.566	113.161
HR 4	93.93	14.37	73.53	9.10	NA	NA	4.65	28	<0.001	1.696	273.584
							*F*	*df*	*p*	*η* ^2^	BF_incl_
Fatigue 1	1.33	0.98	1.07	1.28	0.93	0.80	0.58	2, 42	0.565	0.027	0.246
Fatigue 2	2.01	1.16	1.60	1.18	2.00	1.20	0.67	2, 42	0.509	0.032	0.265
Fatigue 3	3.67	1.35	2.87	1.77	3.067	1.49	1.09	2, 42	0.345	0.049	0.351
Fatigue 4	1.07	1.03	0.68	0.90	0.68	0.62	1.06	2, 42	0.345	0.048	0.315
Fatigue 5	4.13	1.55	3.73	1.71	3.33	1.54	0.93	2, 42	0.401	0.043	0.344

Notes—HEG*:* high-intensity exercise group; LEG*:* low-intensity exercise group; CG: inactive control group; HR: heart rate; Fatigue: subjective fatigue of the performing hand (scale from 0 to 10). HR 1 + Fatigue 1: before baseline; HR 2: 3 min after acute exercise; HR 3 + Fatigue 2: after practice session, immediately after intervention; HR 4 + Fatigue 3: after practice session, 30 min after intervention; Fatigue 4 + 5: before and after practice session, 24 h after intervention. BF_10:_ Bayes factor to quantify the evidence for the alternative hypothesis relative to the null hypothesis. BF_incl:_ Bayes factor to quantify the evidence for ANOVA effects.

**Table 4:** *F* and Bayes statistics of the 3 × 4 RM-ANOVA of *α*-TRPow and *β*-TRPow for each electrode. Statistically significant effects (adjusted α = 0.008) and evidence in favor of the presence of an effect (BF*_incl_*) are marked in bold.

*α*-TRPow	TIME						GROUP						TIME × GROUP					
Electrode	*F*	*df*	*p*	η^2^_p_	η^2^_G_	BF*_incl_*	*F*	*df*	*p*	η^2^_p_	η^2^_G_	BF*_incl_*	*F*	*df*	*p*	η^2^_p_	η^2^_G_	BF*_incl_*
F3	6.05	3, 126	**0.004**	0.126	0.068	**45.499**	0.25	2, 42	0.784	0.012	0.006	0.163	0.63	6, 126	0.631	0.029	0.015	0.069
C3	5.70	3, 126	**0.006**	0.119	0.025	**20.799**	0.25	2, 42	0.784	0.012	0.013	0.386	1.61	6, 126	0.186	0.071	0.014	0.299
CP3	3.89	3, 126	0.022	0.085	0.016	2.629	2.28	2, 42	0.115	0.098	0.082	1.014	1.46	6, 126	0.220	0.065	0.012	0.262
F4	2.27	3, 126	0.110	0.051	0.029	0.451	0.51	2, 42	0.603	0.024	0.011	0.168	1.24	6, 126	0.300	0.056	0.032	0.206
C4	3.52	3, 126	0.043	0.077	0.034	1.624	2.61	2, 42	0.086	0.110	0.068	1.009	1.95	6, 126	0.122	0.085	0.037	0.710
CP4	1.90	3, 126	0.156	0.043	0.014	.245	3.27	2, 42	0.048	0.135	0.097	1.719	2.17	6, 126	0.078	0.094	0.031	1.045
***β*-TRPow**																		
**Electrode**	***F***	***df***	***p***	**η^2^_p_**	**η^2^_G_**	**BF*_incl_***	***F***	***df***	***p***	***η^2^_p_***	***η^2^_G_***	**BF*_incl_***	***F***	***df***	***p***	***η^2^_p_***	***η^2^_G_***	**BF*_incl_***
F3	4.09	3, 126	0.021	0.089	0.040	**4.170**	0.53	2, 42	0.592	0.025	0.014	0.236	0.612	6, 126	0.649	0.028	0.012	0.066
C3	1.26	3, 126	0.287	0.029	0.014	0.125	0.02	2, 42	0.983	0.001	< 0.001	0.144	1.82	6, 126	0.135	0.080	0.039	0.581
CP3	2.48	3, 126	0.090	0.056	0.027	0.494	3.19	2, 42	0.051	0.132	0.075	1.370	2.01	6, 126	0.100	0.087	0.042	0.792
F4	0.90	3, 126	0.443	0.021	0.010	0.089	0.09	2, 42	0.912	0.004	0.002	0.166	0.29	6, 126	0.920	0.014	0.006	0.036
C4	3.04	3, 126	0.053	0.067	0.031	0.905	0.77	2, 42	0.468	0.036	0.020	0.258	2.33	6, 126	0.062	0.100	0.047	1.445
CP4	2.86	3, 126	0.061	0.064	0.034	0.609	4.15	2, 42	0.023	0.165	0.086	2.241	3.90	6, 126	**0.005**	0.157	0.088	**31.463**

## Data Availability

The data presented in this study are available upon request from the corresponding author.

## Supplementary Materials

The following are available online at www.mdpi.com/article/10.3390/ijerph182312512/s1: Table S1: Descriptive results of FM task performance; Table S2: Descriptive results of *α*-TRPow; Table S3: Descriptive results of *β*-TRPow.
