# Supplementary material for: A Single Bout of High-Intensity Cardiovascular Exercise Does Not Enhance Motor Performance and Learning of a Visuomotor Force Modulation Task, but Triggers Ipsilateral Task-Related EEG Activity
Source: Int J Environ Res Public Health. 2021 Nov 27;18(23):12512. doi: 10.3390/ijerph182312512 (PMC8657224; doi:10.3390/ijerph182312512)
Supplement: Supplementary file 1 [file ijerph-18-12512-s001.zip › ijerph-1477522-supplementary.pdf]

Supplementary Materials (Pixa et al., 2021)

Table S1. Descriptive results of FM task performance (mean RMSE ± *SD*) for each group at each analyzed FM task block.

Group	*B-block*	*MP-block*	*iML-block*	*sMM-block*	*lMM-block*
HEG	1.02 ±0.195	0.855 ±0.139	0.872 ±0.195	0.748 ±0.118	0.68 ±0.123
LEG	1.05 ±0.165	0.892 ±0.121	0.836 ±0.135	0.743 ±0.106	0.685 ±0.111
CG	0.984 ±0.184	0.857 ±0.14	0.837 ±0.158	0.73 ±0.126	0.695 ±0.125

Table S2. Descriptive results of α-TRPow (mean ± *SD*) for each group at each FM task block.

Group	Electrode	*B-block*	*MP-block*	*iML-block*	*sMM-block*
	F3				
HEG		0.49 ±1.43	-0.60 ±1.66	-0.37 ±1.65	-0.24 ±1.19
LEG		0.19 ±1.14	-0.16 ±1.92	-0.76 ±2.43	-0.82 ±1.45
CG		0.28 ±1.00	-0.55 ±0.96	-1.00 ±0.99	-0.47 ±1.49
	C3				
HEG		-2.59 ±1.79	-4.13 ±2.79	-4.03 ±2.69	-2.86 ±2.06
LEG		-3.76 ±3.18	-4.47 ±3.33	-4.18 ±3.40	-4.15 ±2.86
CG		-2.67 ±2.14	-3.88 ±3.10	-3.64 ± 3.18	-4.29 ±2.64
	CP3				
HEG		-3.36 ±2.17	-4.81 ±2.96	-4.64 ±2.95	-3.62 ±2.60
LEG		-3.74 ±3.54	-4.89 ±3.52	-4.61 ±3.60	-4.54 ±3.36
CG		-2.21 ±1.94	-2.50 ±2.70	-2.28 ±2.82	-3.00 ±2.52
	F4				
HEG		0.37 ±1.20	-0.72 ±1.29	-0.70 ±1.63	-0.27 ±2.01
LEG		-0.67 ±0.70	-0.18 ±1.56	-0.40 ±1.87	-0.53 ±1.21
CG		0.35 ±1.78	-0.09 ±1.21	-1.30 ±1.13	-0.16 ±1.10
	C4				
HEG		-1.73 ±2.43	-3.40 ±3.29	-3.32 ±2.69	-0.72 ±3.37
LEG		-2.64 ±2.34	-3.43 ±2.99	-3.60 ±3.20	-2.66 ±2.32
CG		-1.37 ±1.63	-1.21 ±2.43	-1.37 ±2.29	-1.54 ±2.67
	CP4				
HEG		-2.89 ±2.53	-4.04 ±2.44	-4.01 ±2.91	-1.98 ±3.30
LEG		-3.26 ±2.43	-4.23 ±3.51	-4.12 ±3.43	-3.46 ±2.74
CG		-2.22 ±1.40	1.34 ±2.85	-1.28 ±2.97	-1.71 ±3.14

Table S3. Descriptive results of β-TRPow (mean ± *SD*) for each group at each FM task block.

Group	Electrode	*B-block*	*MP-block*	*iML-block*	*sMM-block*
	F3				
HEG		-0.318 ±1.93	-1.09 ±1.95	-0.958 ±1.82	0.225 ±2.72
LEG		-0.176 ±1.65	-0.497 ±1.03	-1.18 ±1.52	-0.397 ±1.91
CG		-0.403 ± 1.99	-1.31 ±1.82	-1.54 ±2.88	-0.919 ±2.12
	C3				
HEG		-1.23 ± 1.46	-2.07 ±1.93	-2.24 ±1.94	-1.29 ±1.94
LEG		-1.95 ±2.15	-1.54 ±1.17	-1.88 ±2.14	-1.10 ±1.98
CG		-0.985 ±1.38	-1.64 ±2.40	-1.74 ±2.03	-2.26 ±1.23
	CP3				
HEG		-1.52 ±1.35	-2.30 ±1.62	-2.47 ±1.68	-1.47 ±1.62
LEG		-1.88 ±1.22	-1.88 ±1.10	-2.11 ±1.23	-1.73 ±1.67
CG		-0.633 ±0.7	1.01 ±1.31	-1.24 ±1.32	-1.66 ±0.87
	F4				
HEG		-0.015± 2.22	-0.362 ±1.54	-0.273 ±1.61	0.17 ±2.54
LEG		0.451 ±1.16	0.114 ±0.969	-0.079 ±1.39	-0.165 ±1.39
CG		0.443 ±1.87	-0.044 ±1.94	-0.172 ±3.03	-0.023 ±1.65
	C4				
HEG		-1.27 ±1.82	-1.97 ±2.27	-1.98 ±1.91	-0.46 ±2.06
LEG		-1.78 ±1.49	-2.01 ±1.14	-2.10 ±1.38	-1.05 ±1.83
CG		-0.795 ±1.08	-1.06 ±1.81	-1.19 ±1.51	-1.61 ±1.51
	CP4				
HEG		-1.08 ±1.41	-2.16 ±1.65	-2.22 ±1.40	-0.786 ±1.42
LEG		-1.77 ±1.08	-2.02 ±1.04	-2.07 ±1.08	-1.17 ±1.69
CG		-0.526 ±1.34	-0.837 ±1.37	-0.437 ±1.55	-1.45 ±1.22
